# Dietary Nutrients Involved in One-Carbon Metabolism and Colonic Mucosa-Associated Gut Microbiome in Individuals with an Endoscopically Normal Colon

**DOI:** 10.3390/nu11030613

**Published:** 2019-03-13

**Authors:** Shawn Gurwara, Nadim J. Ajami, Albert Jang, Frances C. Hessel, Liang Chen, Sarah Plew, Zhensheng Wang, David Y. Graham, Clark Hair, Donna L. White, Jennifer Kramer, Themistoklis Kourkoumpetis, Kristi Hoffman, Rhonda Cole, Jason Hou, Nisreen Husain, Maria Jarbrink-Sehgal, Ruben Hernaez, Fasiha Kanwal, Gyanprakash Ketwaroo, Rajesh Shah, Maria Velez, Yamini Natarajan, Hashem B. El-Serag, Joseph F. Petrosino, Li Jiao

**Affiliations:** 1 Department of Medicine, Baylor College of Medicine, Houston, TX 77030, USA; sg17@bcm.edu (S.G.); ajang@bcm.edu (A.J.); frances.hessel@bcm.edu (F.C.H.); liangc@bcm.edu (L.C.); sarah.plew@bcm.edu (S.P.); chestnut890123@gmail.com (Z.W.); dgraham@bcm.edu (D.Y.G.); hair@bcm.edu (C.H.); dwhite1@bcm.edu (D.L.W.); jkramer@bcm.edu (J.K.); themistoklis.kourkoumpetis@bcm.edu (T.K.); rhondac@bcm.edu (R.C.); jkhou@bcm.edu (J.H.); nisreenh@bcm.edu (N.H.); maria.jarbrink-sehgal@bcm.edu (M.J.-S.); ruben.hernaez@bcm.edu (R.H.); kanwal@bcm.edu (F.K.); gyanprakash.ketwaroo@bcm.edu (G.K.); rajeshs@bcm.edu (R.S.); velez.mariaeugenia@va.gov (M.V.); ynataraj@bcm.edu (Y.N.); hasheme@bcm.edu (H.B.E.-S.); 2 The Alkek Center for Metagenomics and Microbiome Research, Department of Molecular Virology and Microbiology, Baylor College of Medicine, Houston, TX 77030, USA; nadimajami@gmail.com (N.J.A.); Kristi.Hoffman@bcm.edu (K.H.); jpetrosi@bcm.edu (J.F.P.); 3 Center for Innovations in Quality, Effectiveness and Safety, Michael E. DeBakey VA Medical Center, Houston, TX 77030, USA; 4 Section of Gastroenterology, Michael E. DeBakey VA Medical Center, Houston, TX 77030, USA; 5 Texas Medical Center Digestive Disease Center, Houston, TX 77030, USA; 6 Dan L Duncan Comprehensive Cancer Center, Baylor College of Medicine, Houston, TX 77030, USA; 7 Center for Translational Research on Inflammatory Diseases (CTRID), Michael E. DeBakey VA Medical Center, Houston, TX 77030, USA

**Keywords:** microbiota, 1-carbon metabolism, methylation, diet, folate, vitamin B, mucosa, *Faecalibacterium*, mucosa

## Abstract

One carbon (1C) metabolism nutrients influence epigenetic regulation and they are supplied by diet and synthesized by gut microbiota. We examined the association between dietary consumption of methyl donors (methionine, betaine and choline) and B vitamins (folate, B2, B6, and B12) and the community composition and structure of the colonic mucosa-associated gut microbiota determined by 16S rRNA gene sequencing in 97 colonic biopsies of 35 men. We used the food frequency questionnaire to assess daily consumption of nutrients, and the UPARSE and SILVA databases for operational taxonomic unit classification. The difference in bacterial diversity and taxonomic relative abundance were compared between low versus high consumption of these nutrients. False discover rate (FDR) adjusted *p* value < 0.05 indicated statistical significance. The bacterial richness and composition differed significantly by the consumption of folate and B vitamins (*p* < 0.001). Compared with higher consumption, a lower consumption of these nutrients was associated with a lower abundance of *Akkermansia* (folate), *Roseburia* (vitamin B2), and *Faecalibacterium* (vitamins B2, B6, and B12) but a higher abundance of *Erysipelatoclostridium* (vitamin B2) (FDR *p* values < 0.05). The community composition and structure of the colonic bacteria differed significantly by dietary consumption of folate and B vitamins.

## 1. Introduction

One carbon (1C) metabolism is a universal metabolic process that serves to activate and transfer 1C units for biosynthesis of purine, thymidine, and homocysteine remethylation [1]. Because riboflavin (vitamin B2), folate (vitamin B9) and cobalamin (vitamin B12) cannot be produced by humans, these vitamins must be supplied via diet. Dietary deficiency in vitamin B can perturb the regulatory network of 1C metabolism and has been associated with increased risk of developing colorectal cancer (CRC) [2,3].

Gut microbiota have been shown to play an important role in CRC [4,5,6]. Gut microbiota synthesize choline, thiamin (vitamin B1), vitamin B2, nicotinic acid (vitamin B3), pantothenic acid (vitamin B5), pyridoxine (vitamin B6), biotin (vitamin B7), folate, and B12 [7,8]. Prior studies have shown an increased requirement for dietary vitamins K, B1, B6, B7, B9 and B12 to maintain health in germ-free mice [9,10]. Systemic research assessed the genomes of 256 common human-gut bacteria for the presence of the biosynthesis pathways for eight B-vitamins. Bacteroidetes, Fusobacteria, and Proteobacteria were identified as the major bacteria with biosynthesis capacity for vitamins B2, B6, B9, and B12 [7]. These microorganisms interact with histones to regulate gene expression [11,12,13] or help absorb or excrete minerals, such as zinc, selenium, and cobalt, which are cofactors of enzymes involved in epigenetic processes [14].

We hypothesized that dietary consumption of nutrients supporting 1C metabolism is associated with the bacteria that rely on or synthesize B vitamins. Therefore, we conducted a cross-sectional study to examine the association between the community composition and structure of the colonic mucosa-associated gut microbiome and dietary consumption of methionine, betaine, choline, folate, and vitamins B2, B6 and B12 in individuals with an endoscopically normal colon.

## 2. Methods

### 2.1. Study Participants

In this cross-sectional study, we prospectively and consecutively recruited male veterans presenting for elective colonoscopy procedures at the Michael E. DeBakey VA Medical Center (MEDVAMC) in Houston between July 2013 and April 2017. A total of 32% of the study participants were referred for a colonoscopy because of occult blood in feces. Individuals were eligible to participate in our study if they were between 50 and 75 years of age. We excluded participants with history of: (1) hereditary polyposis syndromes such as familial adenomatous polyposis and hereditary non-polyposis colon cancer; (2) inflammatory bowel disease; (3) invasive cancer except for non-melanoma skin cancer; (4) colorectal polyps in the past three years; (5) end-stage renal disease requiring dialysis; (6) severe mental disabilities; (7) hospitalization within the past year; (8) oral or systemic use of antibiotics in the past three months; (9) hepatitis B or C infections; (10) HIV or methicillin-resistant *Staphylococcus aureus*-positive infection; or (11) contraindications to obtaining mucosal biopsies. Participants who had changed their dietary habits in the past three months were also excluded. Individuals were eligible for this analysis if they had an endoscopically normal colon.

### 2.2. Data Collection

Participants attended a colonoscopy educational session one to two weeks prior to their colonoscopy, during which a trained research coordinator obtained their informed consent and administered a questionnaire on lifestyle, social history, and medical history. We obtained anthropometric measurements (height, weight, waist, and hip circumference) using a calibrated scale. We assessed food consumption history in the past 12 months using the self-administered validated Block Food Frequency Questionnaire (FFQ) 2005. Participants completed the FFQ survey and mailed it back using pre-paid and pre-addressed envelopes with the aid of a reminder call. The response rate of FFQs was 87%. There were no missing responses in the FFQ. Overall dietary quality was evaluated using the healthy eating index (HEI) 2005 [15].

### 2.3. Colonoscopy and Biopsy Acquirement

Each participant drank one gallon of polyethylene glycol the night before the colonoscopy. Participants were also advised to stop taking aspirin, anti-inflammatory drugs, blood thinners, iron or vitamins with iron seven days before the procedure and to stop medications for diabetes one day before the procedure. All procedures were performed under conscious sedation and involved examination of the entire colon up to the cecum.

Colonic mucosal samples were collected with cold-biopsy forceps introduced through a scope-operating-channel. Biopsies were taken from each colonic segment (cecum, ascending, transverse, descending, sigmoid colons or rectum). All biopsies were immediately placed in a sterile tube on dry ice and subsequently transferred to an −80 °C freezer within 15 min.

After inclusion and exclusion criteria, we enrolled 612 eligible study participants, of which 169 were polyp-free; 131 participants consented to provide a colonic mucosal biopsy, and we sent biopsy samples from 68 participants for microbiome profiling as a part of a case-control study on the risk of advanced adenoma. Among this group, 40 returned the FFQ. Five study participants were further excluded because they had self-reported energy consumption <800 or >5000 kcal per day. A total of 99 mucosa samples were collected from 35 participants. Two biopsies with low sequencing count from two participants were excluded from the final analysis. We, therefore, included 97 mucosa samples from 35 participants in the present analysis. We were not able to get biopsies from six segments from all participants.

The study protocol was approved by the Institutional Review Board of Baylor College of Medicine (BCM) and MEDVAMC.

### 2.4. Microbial DNA Extraction and the 16S rRNA Gene Sequencing

Bacterial sequence analysis was performed at the Alkek Center for Metagenomics and Microbiome Research (CMMR) at BCM. Bacterial genomic DNA was extracted using the MO BIO PowerLyzer UltraClean Tissue & Cell DNA Isolation Kits (MO BIO Laboratories, Clardad, CA, USA). All DNA samples were stored at −80 °C until further analysis.

The 16S rRNA variable region 4 (V4) was amplified by PCR, using the barcoded Illumina adaptor-containing primers 515F and 806R and sequenced in the MiSeq platform (Illumina, San Diego, CA, USA) using the 2 × 250 bp paired-end protocol yielding pair-end reads that overlap almost completely [16,17,18]. The primers used for amplification contain adapters for the MiSeq sequencing and single-index barcodes so that the PCR products may be pooled and sequenced directly [16]. The samples were rarefied to 1648 reads resulting in the loss of all negative controls for the DNA extraction kit. Rarefication ensured that we sampled the most microbial diversity.

### 2.5. Bioinformatics and Taxonomic Assignment

We used the CMMR 16S rRNA gene analysis pipeline for data analysis. The read pairs were demultiplexed based on the unique molecular bar codes, and reads were merged using USEARCH v7.0.1090 [19]. Merging allowed zero mismatches and a minimum overlap of 50 bases, and merged reads were trimmed at the first base with a quality score ≤5. A quality filter was applied to the resulting merged reads and those containing above 0.5% expected errors were discarded. The 16S rRNA gene sequences were clustered into Operational Taxonomic Units (OTUs) at a similarity cutoff value of 97% using the UPARSE algorithm [20]. The chimeras were removed using the UCHIME [21]. The OTUs were mapped to an optimized version of the SILVA database containing only the sequences from the V4 region of 16S RNA to determine taxonomies [22]. A rarefied OTU table was constructed from the output files generated in the previous two steps. We used the ATIMA (Agile Toolkit for Incisive Microbial Analyses) software to perform the downstream analyses [23,24].

### 2.6. Statistical Analysis

The bacterial alpha-diversity (richness using the observed OTUs; richness and evenness using the Shannon Index), beta-diversity (dissimilarity in community composition), and the taxa abundance (mainly at the phylum and the genus levels) were assessed according to the consumption of 1C metabolism nutrients [25]. We categorized high- versus low-consumption of certain nutrients based on the median value of the daily consumption in our study population (169 mg for total choline, 95 mg for betaine, 0.80 mg for methionine, 227 mcg for dietary folate equivalent, 1.04 mg for vitamin B2, 0.87 mg for vitamin B6, and 2.38 mcg for vitamin B12). The dietary folate equivalent value used in this study included both folate naturally present in foods and fortified folic acid. We examined all OTU with the relative abundance >0.1%.

Sociodemographic and clinical characteristics of participants were compared according to low or high consumption of vitamin B2 (as an example), using the student’s *t* test or χ^2^ test. To evaluate and visualize the beta-diversity, principal coordinate analysis (PCoA) plots were constructed using the weighted UniFrac distance matrix [26] and the Monte Carlo permutation test was performed to estimate the *p* values. The relative abundances of bacteria by high versus low consumption were compared using the Mann–Whitney test. We also used the univariate linear regression model to examine the association between the relative abundance of the major bacteria phylum or genus and nutrient intake (dependent variable). Folate from food, folic acid, and free choline was also used as the dependent variable in this linear regression analysis.

Major bacteria with significant findings in both dichotomous and linear regression were further assessed using the multivariate analysis. To account for potential confounding factors, based on the negative binomial distribution of sequence count, we used the R package DESeq2 to estimate fold change (FC) coefficients of the relative bacterial counts and the dispersion parameters between low versus high consumption with adjustment for age, ethnicity, smoking status, alcohol consumption, body mass index (BMI), diabetes, and hypertension. The cluster ID was adjusted to account for dependent sequencing data from different segments from the same participant. All *p*-values were adjusted for multiple comparisons with the False Discovery Rate (FDR) algorithm in the microbiome analyses [27]. Less common bacteria were not run for linear regression, but were run for multivariate analysis.

All statistical analyses were performed using SAS 9.4 (SAS Inc., Cary, NC, USA) and R statistical software (version 3.4.4, R foundation). All tests were two-sided, and *p* values < 0.05 for the general analysis and FDR *p* values < 0.05 for the microbiome analysis indicated statistical significance.

## 3. Results

The study analysis involved 35 men, 71% of whom were non-Hispanic white. A total of 97 mucosa biopsies were included in the analysis. The bacterial compositions were similar between different segments (Figure S1). The general characteristics of these participants were summarized based on high or low dietary consumption of vitamin B2 (Table 1). There was no significant difference in age, ethnicity, BMI, overall dietary quality, or mucosal sample location between two groups. The high-vitamin-B2-consumption group had an insignificantly higher HEI score than the low consumption group (*p* = 0.08).

**Figure 1:**
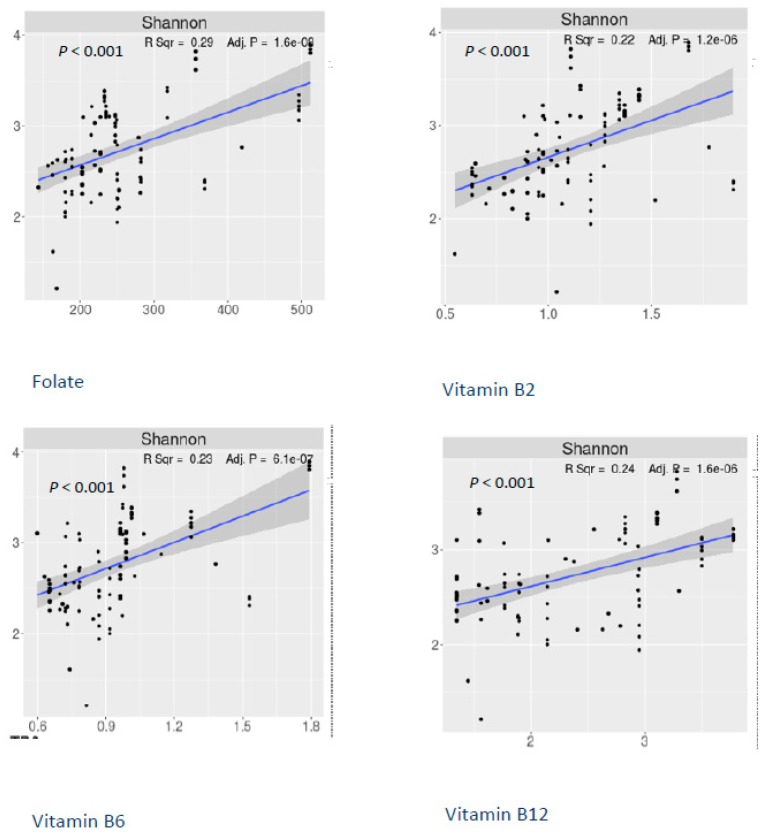
Alpha-diversity (Shannon index) by dietary intake of vitamins B2, B6, B12, and folate.

**Figure 2:**
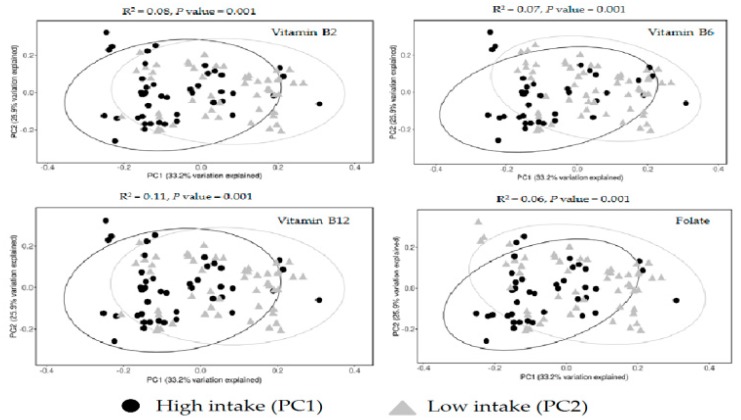
Beta-diversity by dietary intake of vitamins B2, B6, B12, and folate.

A higher dietary consumption of folate and vitamins B2, B6 and B12 was associated with a significantly greater richness and evenness of the gut microbiota (Shannon index *p* < 0.001, Figure 1). Beta diversity also differed significantly by high or low consumption of these nutrients (weighted Unifrac *p* value < 0.005, Figure 2). However, dietary consumption of choline, methionine, or betaine was not significantly associated with alpha and beta diversity of gut microbiota.

**Figure 3:**
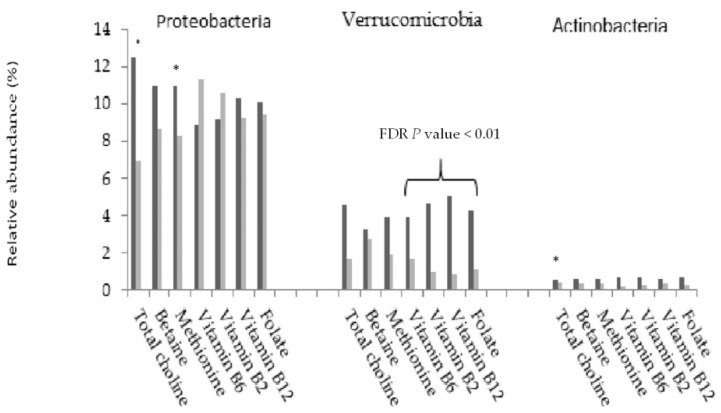
Relative abundance of Proteobacteria, Verrucomicrobia, and Actinobacteria by dietary consumption of one-carbon metabolism nutrients (dark gray bar: high intake; light gray bar: low intake). * FDR-*p* value < 0.05.

At the phylum level, a higher dietary consumption of total choline and methionine was significantly associated with a lower relative abundance of Firmicutes and a higher abundance of Proteobacteria than lower consumption. Participants with a higher consumption of folate and vitamins B2, B6, and B12 had significantly higher abundance of Verrucomicrobia (FDR *p* values < 0.05) (Figure 3 and Table S1). Actinobacteria was not associated with dietary 1C nutrient consumption. Bacterial families that were mostly influenced by B vitamins included Lachnospiraceae, Ruminococcaceae, Enterobacteriacea, Prevoteriaceae, Verrucomicrobiaceae, Porphyromonadaceae, Erysipelotrichaceae, and Rikenellaceae.

At the genus level, the abundance of bacteria did not differ significantly by dietary consumption of total choline and methionine (Table S2). The relative abundance of *Faecalibacterium*, *Roseburia*, *Subdoligranulum*, and *Akkermansia* was significantly higher with higher consumption of folate and vitamins B2, B6, or B12, whereas the relative abundance of *Erysipelatoclostridium* was significantly lower in those with higher consumption of folate, vitamins B2, B6, and B12 and betaine. The abundance of *Bacteroides* was significantly lower with higher consumption of vitamin B12. The abundance of *Escherichia/Shigella* was significantly lower with higher consumption of vitamins B2 and B6. In addition, we found higher relative abundance of multiple unclassified genera of the Lachnospiraceae and Ruminococcaceae families in those who had higher consumption of dietary methyl donor and B vitamins. However, one genus, *Lachnospiraceae (Unco8895)*, with a relative abundance of 6%, was significantly less abundant in participants with higher consumption of vitamin B nutrients (Table 2).

The relative abundance of several uncommon bacteria genera (relative abundance <2%), *Alistipes*, *Dialister*, and *Odoribacter*, was significantly higher in those with higher consumption of folate and vitamins B2, B6 and B12. *Bifidobacterium* was significantly more abundant with higher consumption of vitamin B6 (Table 2).

The linear regression also showed a positive association between the consumption of vitamin B nutrients and *Faecalibacterium* and *Alistipes*, and an inverse association with *Lachnospiraceae (uncO8895)* (Table 3). In addition, folate from foods was significantly inversely associated with *Bacteroides* and positively associated with *Roseburia* (*R*^2^ = 0.10, FDR *p* = 0.008 for both); folic acid fortification was insignificantly positively associated with *Faecalibacterium* and *Subdoligranulum* (*R*^2^ = 0.07, FDR *p* = 0.057 for both). Total choline was significantly positively associated with *Akkermansia* (*R*^2^ = 0.11, FDR *p* = 0.009) and free choline was significantly positively associated with *Escherichia* (*R*^2^ = 0.12, FDR *p* = 0.007).

The multivariable analyses confirmed a significant association between higher consumption of total choline and methionine and higher Proteobacteria and lower Firmicutes, as well as a significant positive association between folate and Verrucomicrobia. At the genus level, higher consumption of vitamin B2 was positively associated with *Faecalibacterium*, *Odoribacter*, and *Dialister*, but inversely associated with *Erysipelatoclostridium*; vitamin B6 and folate were positively associated with *Odoribacter* and *Dialister*, and vitamin B12 was positively associated with *Faecalibacterium* and *Dialister* (Table 4). 

## 4. Discussion

Our study showed that dietary B vitamins involved in 1C metabolism were associated with variations in the richness, composition, and abundance of the colonic mucosa-associated microbiome in participants with an endoscopically normal colon. The higher consumption of folate and vitamins B2, B6 or B12 was, for the most part, associated with more abundant *Faecalibacterium*, *Alistipes*, and *Odoribacter*, but less abundant *Bacteroides*, *Erysipelatoclostridium*, and *Lachnospiraceae (Unc8895)*. The Clostridia class of the Firmicutes phylum was most influenced by the 1C metabolism nutrients.

Our study found little evidence for an alteration of the colonic gut microbiota according to dietary intake of methionine and betaine. We showed a significant increase in Proteobacteria but a decrease in Firmicutes in individuals who had higher consumption of total choline. A higher dietary total choline intake has been associated with increased risk of CRC in women [28]. Animal studies have shown the protective roles of dietary methyl donor depletion in intestinal adenoma development in Apc(Min/+) mice [29]. Several studies also showed positive associations between serum choline levels and increased risk of CRC [30,31,32]. Microbial metabolism of choline may contribute to the risk of atherosclerosis by supporting increased microbial production of trimethylamine and then trimethylamine-N-oxide [33]. An increased abundance of Proteobacteria and its constituent family Enterobacteriaceae has been found in the biopsies from patients with colonic adenocarcinoma versus adenoma and healthy tissue [34]. We found free choline to be significantly positively associated with the abundance of *Escherichia* (Enterobacteriaceae family). Overall, our microbiome study would support an adverse effect of higher consumption of choline in humans.

Two genera within the Firmicutes phylum, *Faecalibacterium* and *Roseburia*, are known as butyrate-producing, gram-positive anaerobic bacteria and have been shown to be associated with a decreased risk of several chronic diseases [35,36,37,38]. Two metagenome studies have shown a significant inverse association between *Roseburia* and risk of diabetes [30,36]. A lower abundance of *Faecalibacterium* has been reported in the stool of CRC patients [38]. In our study, these bacteria were more abundant in participants with a higher consumption of dietary vitamin B. Vitamins B2, B6, and B12 may maintain or promote the growth of anti-inflammatory, butyrate-producing commensals in the gut. Butyrate is a short-chain fatty acid (SCFA) that is a potent inhibitor of histone deacetylases [11,39]. SCFAs have been shown to regulate epigenetic programming in various tissues, including the proximal colon [40]. Therefore, these bacteria may modulate epigenetic events through butyrate or other SCFAs.

We observed an increased abundance in *Akkermansia* among those with a higher consumption of 1C metabolism B vitamins, although only the association with folate was significant. *Akkermansia* is possibly beneficial through anti-inflammatory and beneficial metabolic mechanisms [41,42]. A higher abundance of *Akkermansia* has been associated with seizure prevention in epilepsy, better response to chemotherapy, and being non-diabetic in mice [42,43]. Because folate may track with the consumption of other nutrients, we cannot exclude the possibility that the observed association with the gut microbiome is attributable to the consumption of other nutrients that can modulate *Akkermansia*.

Several less common bacteria were also influenced by B vitamins. *Alistipes* and *Odoribacter*, both members of the Bacteroidetes phylum, were significantly increased in individuals with higher consumption of 1C metabolism B vitamins. *Odoribacter splanchnus* is a known producer of acetate, propionate and butyrate. Decreased *Odoribacter* may affect host inflammation via reduced SCFA [44]. A recent study showed that *Alistipes* and *Odoribacter* can produce sulfonolipids in mice fed a high-fat diet and indicated that these bacteria can affect lipid metabolism [44]. *Dialister* is an opportunistic genus that belongs to the Firmicutes phylum and Clostridia class, and it is more abundant with higher consumption of B vitamins. *Dialister* is one of the bacteria enriched by whole-grain consumption and has been associated with reduced levels of interleukin-6 in one clinical trial in 28 healthy individuals. *Dialister* may condition the capacity to be immunologically responsive to whole grain [45]. *Alistipes*, *Dialister*, and *Odoribacter* are, therefore, potentially beneficial in the context of 1C metabolism B vitamin consumption.

On the other hand, we found that the abundance of *Erysipelatoclostridium* was significantly decreased in individuals with higher consumption of vitamin B2. Congruent negative associations between *Erysipelatoclostridium* and several health conditions have been established. *Erysipelatoclostridium* was enriched in the feces of humans with gout and metabolic syndrome [46,47,48]. In addition, a common unclassified genus *Lachnospiraceae (Unco8895)*, was significantly lower in individuals with higher consumption of all the B vitamins investigated. A further metagenomics whole-genome shotgun sequencing study is required to identify the unclassified species and the functions related to these and other taxa.

Previous studies have shown that Bacteroidetes, Fusobacteria and Proteobacteria are major producers of B vitamins. The major genus of Bacteroidetes, *Bacteroides*, was significantly more abundant in those consuming less vitamin B12. The linear regression analysis also showed its inverse association with the consumption of vitamins B2, B6, and B12. The major genus of Proteobacteria, *Escherichia/Shigella*, was significantly less abundant with higher consumption of vitamins B2 and B6. However, this association was not confirmed in the linear regression and multivariable analysis. We observed only an inverse association between the abundance of Fusobacteria and vitamin B6 in the linear regression. The abundance of Fusobacteria is 2% in our study. Larger studies are needed to further investigate the potential interaction between dietary consumption of 1C metabolism nutrients and the bacteria structure and their biological consequences.

Our analysis of the colonic mucosa-associated gut microbiome provided baseline information on the association between diet/nutrient and gut microbiota in largely healthy individuals. Our findings were less likely to be affected by antibiotic use or medication use per our study design. We controlled for the potential confounding effects of age and BMI by using a multivariable analysis. The contribution of multiple biopsies from participants was also adjusted. Because dietary information was collected before the colonoscopy, a recall bias was less likely. However, we found that those who returned the FFQ were more likely to be white and young, but had a lower HEI. Therefore, a few limitations of the study should be noted. The generalizability of our findings to other populations was limited not only because participants were all middle-aged men, but also because they had lower consumption of the nutrients than the recommended daily allowance (RDA) for middle-aged men, which is 400 mcg for dietary folate equivalent, 1.3 mg for vitamin B2, 1.7 mg for vitamin B6 and 2.4 mcg for vitamin B12 [49]. The median intake in our population was 227 mcg for dietary folate equivalent, 1.04 mg for vitamin B2, 0.81 mg for vitamin B6, and 2.38 mcg for vitamin B12. Second, the comparison of the relative abundance of minor/rare bacteria (<0.1%) may not be as accurate as for the more abundant ones. Third, our assessment of dietary consumption was based on a self-reported, one-time measurement; the possibility of information bias was not excluded. The measurement of biomarkers of these nutrients would have strengthened our investigation and will be pursued in our future study.

## 5. Conclusions

In summary, our study showed that individuals with a lower dietary consumption of 1C metabolism B vitamins had a lower abundance of butyrate-producing commensal gut bacteria, but they had a higher abundance of potentially harmful bacteria. Further studies are required to examine how dietary B vitamins are metabolized by both the host and the microbes and how they contribute to 1C metabolism and epigenetic events symbiotically and to the pathogenesis of various diseases.

## Figures and Tables

**Table 1:** Basic characteristics of the 35 study participants according to dietary vitamin B2 consumption defined by median intake.

Characteristics	High B2 Diet	Low B2 Diet	*p* Value
	(*n* = 17)	(*n* = 18)	
Age, mean (SD)	62.9 (4.8)	61.3 (6.1)	0.40
Gender (Male)	17 (100)	17 (94.4)	0.32
Race			
Non-Hispanic White	13 (76.5)	12 (66.6)	0.60
Hispanic White	1 (5.9)	3 (16.7)	
Black	3 (17.6)	3 (16.7)	
BMI (kg/m^2^), mean (SD)	33.6 (6.5)	33.9 (6.6)	0.87
BMI, categorical			
<30	4 (23.5)	6 (33.3)	0.52
≥30	13 (76.5)	12 (66.7)	
Hypertension	14 (82.4)	12 (66.7)	0.29
Diabetes	9 (52.9)	8 (44.4)	0.62
Smoking status			
Never	9 (52.9)	5 (27.8)	0.30
Past	3 (17.6)	4 (22.2)	
Current	5 (29.4)	9 (50.0)	
Alcohol drinking			
Never	6 (35.3)	2 (11.1)	0.16
Past	5 (29.4)	11 (61.1)	
Current	5 (29.4)	5 (27.8)	
HEI total Scores ^1^	63.3 (9.4)	57.8 (8.6)	0.08
Segments site			
Cecum	10 (18.5)	7 (15.6)	0.92
Ascending	9 (16.7)	9 (20.0)	
Transverse	8 (14.8)	4 (8.9)	
Descending	6 (11.1)	5 (11.1)	
Sigmoid	11 (20.4)	12 (26.7)	
Rectum	10 (18.5)	8 (17.8)	

BMI, Body mass index; HEI, Healthy Eating Index; SD, standard deviation. ^1^ HEI score ranges from 0–100. A higher score indicates a healthier dietary quality.

**Table 2:** Mean relative abundance (%) and its 95% confidence interval of selected bacterial genus by dietary consumption of B vitamins of one-carbon metabolism ^1^.

Nutrient	Genus	Mean Relative Abundance (%) (95% Confidence Interval)		
		High Intake	Low Intake	FDR *p*-Value
Folate	*Faecalibacterium*	10.79 (8.63, 12.95)	6.14 (3.67, 8.60)	0.006
	*Lachnospiraceae (UncO8895)*	3.26 (1.52, 5.00)	9.47 (5.85, 13.09)	<0.001
	*Lachnospiraceae (Unc94789)*	1.89 (1.21, 2.56)	4.04 (2.46, 5.61)	0.025
	*Akkermansia*	4.31 (2.45, 6.18)	1.18 (−0.75, 3.11)	<0.001
	*Subdoligranulum*	2.36 (1.85, 2.88)	1.22 (0.83, 1.60)	0.006
	*Erysipelatoclostridium*	0.96 (0.12, 1.79)	1.92 (0.63, 3.20)	0.002
	*Alistipes*	1.80 (1.22, 2.37)	0.54 (0.20, 0.87)	<0.001
	*Haemophilus*	1.83 (1.15, 2.51)	0.44 (0.08, 0.80)	0.006
	*Desulfovibrio*	1.31 (0.68, 1.94)	0.13 (0.03, 0.22)	0.032
	*Prevotellaceae (Unc04zvf)*	1.05 (0.49, 1.62)	0.31 (−0.13, 0.75)	0.025
	*Odoribacterium*	0.62 (0.45, 0.79)	0.10 (0.04, 0.17)	<0.001
	*Dialister*	0.28 (0.16, 0.40)	0.03 (0, 0.06)	<0.001
Vitamin B2	*Faecalibacterium*	11.32 (9.11, 13.53)	5.92 (3.60, 8.24)	<0.001
	*Lachnospiraceae (UncO8895)*	2.04 (1.09, 2.98)	10.38 (6.78, 13.98)	<0.001
	*Escherichia/Shigella*	3.83 (1.77, 5.89)	6.39 (3.93, 8.84)	0.027
	*Lachnospiraceae (Unc94789)*	1.97 (1.22, 2.73)	3.74 (2.31, 5.17)	0.019
	*Akkermansia*	4.67 (2.7, 6.65)	1.03 (−0.72, 2.78)	<0.001
	*Roseburia*	3.24 (2.38, 4.11)	1.75 (1.03, 2.47)	0.039
	*Lachnoclostridium*	1.51 (0.99, 2.04)	2.32 (1.73, 2.90)	0.034
	*Subdoligranulum*	2.53 (2.00, 3.06)	1.11 (0.76, 1.47)	<0.001
	*Erysipelatoclostridium*	0.14 (0.03, 0.25)	2.82 (1.33, 4.30)	<0.001
	*Alistipes*	1.85 (1.28, 2.43)	0.58 (0.18, 0.99)	<0.001
	*Desulfovibrio*	1.27 (0.64, 1.90)	0.29 (−0.07, 0.64)	0.022
	*Prevotellaceae (Unc04zvf)*	1.13 (0.53, 1.73)	0.28 (−0.12, 0.68)	0.009
	*Barnesiella*	0.88 (0.58, 1.17)	0.30 (0.02, 0.59)	0.042
	*Odoribacterium*	0.61 (0.46, 0.77)	0.16 (0.02, 0.30)	<0.001
	*Dialister*	0.30 (0.18, 0.43)	0.03 (0, 0.06)	<0.001
Vitamin B6	*Faecalibacterium*	10.76 (8.72, 12.80)	5.94 (3.25, 8.62)	0.003
	*Lachnospiraceae (UncO8895)*	5.11 (2.57, 7.64)	6.93 (4.10, 9.76)	0.039
	*Escherichia/Shigella*	3.70 (1.77, 5.63)	7.00 (4.33, 9.66)	0.005
	*Lachnospiraceae (Unc94789)*	1.67 (1.03, 2.32)	4.49 (2.89, 6.09)	<0.001
	*Akkermansia*	3.87 (2.11, 5.63)	1.70 (−0.50, 3.90)	0.010
	*Lachnoclostridium*	1.34 (0.99, 1.69)	2.72 (1.93, 3.51)	0.005
	*Subdoligranulum*	2.25 (1.75, 2.75)	1.33 (0.89, 1.77)	0.024
	*Erysipelatoclostridium*	1.12 (0.22, 2.01)	1.72 (0.50, 2.95)	0.015
	*Alistipes*	1.65 (1.15, 2.14)	0.70 (0.13, 1.27)	<0.001
	*Prevotellaceae (Unc04zvf)*	1.12 (0.54, 1.69)	0.17 (−0.17, 0.51)	0.010
	*Barnesiella*	0.81 (0.55, 1.08)	0.31 (−0.02, 0.64)	0.023
	*Odoribacterium*	0.56 (0.42, 0.71)	0.17 (0, 0.33)	<0.001
	*Dialister*	0.28 (0.16, 0.39)	0.02 (0, 0.05)	<0.001
	*Bifidobacterium*	0.26 (0.11, 0.41)	0.02 (0.01, 0.03)	0.024
Vitamin B12	*Bacteroides*	24.51 (20.46, 28.57)	34.65 (29.76, 39.54)	0.016
	*Faecalibacterium*	12.66 (10.10, 15.22)	4.84 (3.42, 6.26)	<0.001
	*Lachnospiraceae (UncO8895)*	2.39 (1.38, 3.40)	9.47 (5.99, 12.95)	0.026
	*Akkermansia*	5.04 (2.99, 7.10)	0.87 (−0.76, 2.50)	<0.001
	*Roseburia*	3.38 (2.50, 4.27)	1.70 (1.00, 2.39)	0.007
	*Lachnoclostridium*	1.56 (0.99, 2.13)	2.22 (1.67, 2.76)	0.046
	*Erysipelatoclostridium*	0.19 (0.06, 0.32)	2.59 (1.18, 4.00)	0.001
	*Alistipes*	1.85 (1.23, 2.47)	0.67 (0.29, 1.05)	<0.001
	*Haemophilus*	1.84 (1.14, 2.54)	0.63 (0.15, 1.11)	0.026
	*Odoribacterium*	0.53 (0.36, 0.69)	0.28 (0.12, 0.44)	0.026
	*Dialister*	0.31 (0.17, 0.44)	0.04 (0, 0.08)	<0.001

^1^ Genera with the significant difference between high versus low dietary nutrients intake were shown (FDR *p* value < 0.05).

**Table 3:** Linear regression of the relative abundance of genus and nutrients ^1^.

	Folate	Vitamin B2	Vitamin B6	Vitamin B12
	*R* ^2^	*R* ^2^	*R* ^2^	*R* ^2^
**Increase with more consumption**				
*Faecalibacterium*		13%	6%	13%
*Akkermansia*				9%
*Subdoligranulum*	11%			
*Alistipes*	7%	20%	15%	
*Haemophilus*				7%
*Roseburia*		6%		
*Parabacteroides*		8%		
**Decrease with more consumption**				
*Bacteroides*		5%	6%	15%
*Erysipelatoclostridium*		8%		
*Fusobacterium*			7%	
*Lachnospiraceae (UncO8895)*	8%	8%		5%

^1^ Genera with a significant difference between high versus low dietary nutrient intake are shown (FDR *p* value < 0.05).

**Table 4:** Multivariable analysis of mean abundance of phylum and genus by dietary consumption of nutrients (high or low) of one-carbon metabolism.

		Prevalence (%)		Median Relative Count ^1^		
Nutrients	Phylum	High	Low	High	Low	Fold Change (95% CI) ^2^
**Total choline**	*Proteobacteria*	100	100	137	102	**0.63 (0.48–0.83)**
	*Firmicutes*	100	100	752	789	**1.19 (1.02–1.39)**
**Methionine**	*Proteobacteria*	100	100	113	112	**0.66 (0.49–0.91)**
	*Firmicutes*	100	100	752	797	1.42 (1.18–1.71)
**Folate**	*Verrucomicrobia*	76.2	26.5	13.3	0	**0.24 (0.06–0.96)**
**Vitamin B2**	*Verrucomicrobia*	79.2	34.1	21.1	0	0.47 (0.11–1.97)
**Vitamin B6**	*Verrucomicrobia*	70.7	41	17.4	0	0.75 (0.25–2.20)
	**Genus**					
**Vitamin B2**	*Odoribacter*	77.4	38.6	10.4	0	**0.10 (0.05–0.20)**
	*Roseburia*	96.2	86.4	46.6	10.2	**0.24 (0.13–0.42)**
	*Faecalibacterium*	100	86.4	181	38.1	**0.56 (0.32–0.97)**
	*Erysipelatoclostridium*	28.3	70.5	0	4.35	**24.8 (9.25–66.62)**
	*Dialister*	56.6	9.1	1.41	0	**0.06 (0.02–0.18)**
	*Akkermansia*	79.2	34.1	22.6	0	**0.16 (0.03–0.99)**
**Vitamin B6**	*Odoribacter*	72.4	41	8.02	0	**0.28 (0.12–0.61)**
	*Roseburia*	94.8	87.2	42.6	10.7	**0.29 (0.15–0.57)**
	*Faecalibacterium*	100	84.6	170	30.8	0.67 (0.35–1.29)
	*Erysipelatoclostridium*	36.2	64.1	0	2.43	0.91 (0.38–2.18)
	*Dialister*	50	12.8	0.23	0	**0.14 (0.05–0.34)**
	*Akkermansia*	70.7	41	15.9	0	0.65 (0.25–1.71)
**Vitamin B12**	*Odoribacter*	72.9	46.9	9.96	0	0.69 (0.46–1.02)
	*Roseburia*	97.9	85.7	51.9	7.39	**0.42 (0.29–0.60)**
	*Faecalibacterium*	97.9	89.8	220	30.6	**0.49 (0.34–0.71)**
	*Erysipelatoclostridium*	31.3	63.3	0	2.1	**1.35 (1.00–1.83)**
	*Dialister*	58.3	12.2	1.49	0	**0.37 (0.25–0.55)**
	*Akkermansia*	83.3	34.7	26.8	0	0.81 (0.62–1.05)
**Folate**	*Odoribacter*	74.6	32.4	5.26	0	**0.23 (0.12–0.44)**
	*Roseburia*	92.1	91.2	46.1	7.45	1.21 (0.69–2.11)
	*Faecalibacterium*	100	82.4	146	72.6	**0.57 (0.35–0.93)**
	*Erysipelatoclostridium*	38.1	64.7	0	5.24	1.59 (0.75–3.39)
	*Dialister*	49.2	8.8	0	0	**0.19 (0.08–0.48)**
	*Akkermansia*	76.2	26.5	6.55	0	**0.28 (0.09–0.89)**

^1^ Normalized median count calculated by dividing the count by a size factor in DESeq function; ^2^ Coefficients of fold change were estimated by the empirical Bayes shrinkage method based on negative binomial distribution. Model was adjusted for age, race (white vs. non-white), Hispanic (yes vs. no), BMI (<25, 25−<30, ≥30 kg/m^2^), smoking (never, former, or current), alcohol consumption (never, former, or current), type 2 diabetes (yes vs. no), colon segment (ascending, cecum, descending, rectum, sigmoid, or transverse), and cluster ID. The statistically significant differences were bolded.

## Supplementary Materials

The following are available online at https://www.mdpi.com/2072-6643/11/3/613/s1, Figure S1: Beta-diversities of the mucosa-associated gut bacterial communities. Table S1: Mean relative abundance (%) and its 95% confidence interval of major phylum by dietary consumption of nutrients (high or low) of one-carbon metabolism, Table S2: Mean relative abundance (%) and its 95% confidence interval of selected bacterial genus by dietary consumption of methyl donor of one-carbon metabolism.
