# Supplementary material for: Dietary Nutrients Involved in One-Carbon Metabolism and Colonic Mucosa-Associated Gut Microbiome in Individuals with an Endoscopically Normal Colon
Source: Nutrients. 2019 Mar 13;11(3):613. doi: 10.3390/nu11030613 (PMC6470629; doi:10.3390/nu11030613)
Supplement: Supplementary file 1 [file nutrients-11-00613-s001.pdf]

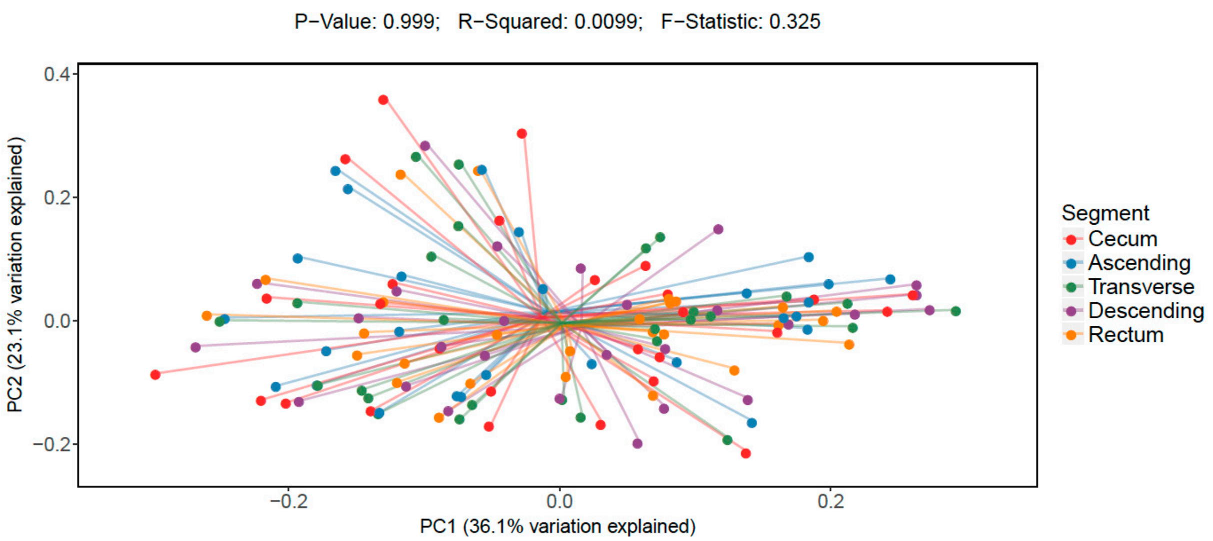

Supplemental figure S1. Beta-diversities of the mucosa-associated gut bacterial communities.

Supplemental table S1. Mean relative abundance (%) and its 95% confidence interval of major phylum by dietary consumption of nutrients (high or low) of one-carbon metabolism[1].

Nutrient	Phylum	Mean relative abundance (%) [95% confidence interval]		
		**High intake**	**Low intake**	**FDR *P*-value**
Total choline	Firmicutes	44.26 [41.00, 47.53]	50.59 [46.42, 54.76]	**0.049**
	Bacteroidetes	35.84 [31.37, 40.30]	37.86 [33.83, 41.9]	0.641
	Proteobacteria	13.16 [10.72, 15.59]	6.95 [4.90, 8.99]	**<0.001**
	Verrucomicrobia	4.58 [2.31, 6.86]	1.67 [0.07, 3.28]	0.091
	Fusobacteria	1.42 [0.43, 2.41]	2.37 [0.54, 4.21]	0.372
	Actinobacteria	0.57 [0.23, 0.90]	0.47 [0.35, 0.60]	**0.028**
Betaine	Firmicutes	44.56 [40.60, 48.52]	50.69 [46.99, 54.39]	0.194
	Bacteroidetes	37.80 [33.13, 42.46]	36.07 [32.29, 39.84]	0.908
	Proteobacteria	10.98 [8.16, 13.81]	8.69 [6.85, 10.54]	0.501
	Verrucomicrobia	3.29 [1.33, 5.24]	2.77 [0.79, 4.74]	0.464
	Fusobacteria	2.66 [0.67, 4.64]	1.22 [0.31, 2.13]	0.501
	Actinobacteria	0.63 [0.32, 0.94]	0.41 [0.28, 0.54]	0.784
Methionine	Firmicutes	44.48 [40.85, 48.12]	51.48 [47.49, 55.46]	**0.012**
	Bacteroidetes	39.10 [34.89, 43.31]	34.30 [30.19, 38.40]	0.234
	Proteobacteria	11.11 [8.97, 13.25]	8.28 [5.64, 10.92]	**0.012**
	Verrucomicrobia	3.93 [1.90, 5.96]	1.93 [0.14, 3.73]	0.587
	Fusobacteria	0.66 [0.13, 1.18]	3.47 [1.22, 5.71]	0.870
	Actinobacteria	0.60 [0.31, 0.89]	0.42 [0.30, 0.54]	0.268
Folate	Firmicutes	47.71 [44.46, 50.96]	47.58 [42.67, 52.49]	0.856

	Bacteroidetes	36.12 [32.18, 40.06]	38.07 [33.46, 42.67]	0.730
	Proteobacteria	10.12 [7.88, 12.35]	9.42 [6.82, 12.01]	0.730
	Verrucomicrobia	4.31 [2.45, 6.18]	1.18 [-0.75, 3.11]	**<0.001**
	Fusobacteria	0.91 [0.25, 1.56]	3.39 [0.97, 5.81]	0.856
	Actinobacteria	0.67 [0.40, 0.94]	0.31 [0.21, 0.40]	0.554
Vitamin B2	Firmicutes	47.15 [43.71, 50.59]	48.26 [43.75, 52.78]	0.862
	Bacteroidetes	37.74 [33.97, 41.51]	35.94 [31.15, 40.73]	0.862
	Proteobacteria	9.2 [7.05, 11.35]	10.58 [7.89, 13.27]	0.862
	Verrucomicrobia	4.68 [2.70, 6.65]	1.03 [-0.72, 2.78]	**<0.001**
	Fusobacteria	0.38 [0.19, 0.56]	3.8 [1.53, 6.08]	0.862
	Actinobacteria	0.68 [0.39, 0.97]	0.33 [0.24, 0.42]	0.862
Vitamin B6	Firmicutes	49.8 [46.21, 53.39]	44.33 [40.18, 48.48]	0.091
	Bacteroidetes	36.27 [32.61, 39.94]	37.93 [32.81, 43.05]	0.428
	Proteobacteria	8.86 [6.85, 10.87]	11.33 [8.39, 14.27]	0.215
	Verrucomicrobia	3.88 [2.11, 5.64]	1.70 [-0.5, 3.90]	**0.010**
	Fusobacteria	0.37 [0.20, 0.54]	4.35 [1.74, 6.96]	0.428
	Actinobacteria	0.69 [0.43, 0.95]	0.26 [0.17, 0.34]	0.066
Vitamin B12	Firmicutes	48.08 [44.97, 51.2]	47.2 [42.52, 51.88]	0.814
	Bacteroidetes	35.01 [32.17, 37.85]	38.96 [33.61, 44.30]	0.424
	Proteobacteria	10.32 [7.98, 12.65]	9.31 [6.84, 11.77]	0.424
	Verrucomicrobia	5.05 [2.99, 7.11]	0.87 [-0.76, 2.50]	**<0.001**
	Fusobacteria	0.69 [0.13, 1.25]	3.25 [1.14, 5.36]	0.814
	Actinobacteria	0.64 [0.34, 0.94]	0.39 [0.27, 0.51]	0.814

1. The median of the nutrient intake was used to dichotomize low versus high nutrient intake.

Supplemental table S2. Mean relative abundance (%) and its 95% confidence interval of selected bacterial genus by dietary consumption of methyl donor of one-carbon metabolism[1].

**Nutrient**	**Genus**	**Mean relative abundance (%) [95% confidence interval]**		
		**High intake**	**Low intake**	**FDR *P*-value**
Total choline	*Bacteroides*	27.95 [22.54, 33.35]	30.71 [26.65, 34.76]	0.515
	Faecalibacterium	8.42 [5.99, 10.84]	9.27 [6.91, 11.62]	0.718
	Escherichia/Shigella	7.18 [4.59, 9.78]	3.09 [1.27, 4.91]	0.055
	Akkermansia	4.58 [2.30, 6.86]	1.67 [0.07, 3.28]	0.257
	Roseburia	3.29 [2.28, 4.29]	1.95 [1.31, 2.59]	0.257
	Erysipelatoclostridium	1.06 [0.04, 2.09]	1.6 [0.58, 2.63]	0.874
	Odoribacterium	0.30 [0.18, 0.42]	0.50 [0.31, 0.69]	0.669
	Dialister	0.23 [0.10, 0.37]	0.13 [0.05, 0.21]	0.434
	Bifidobacterium	0.23 [0.04, 0.42]	0.11 [0.04, 0.18]	0.176
Betaine	*Bacteroides*	31.29 [26.59, 35.98]	27.60 [22.95, 32.26]	0.427
	Faecalibacterium	7.48 [5.60, 9.36]	10.23 [7.48, 12.99]	0.457
	Escherichia/Shigella	6.05 [3.32, 8.78]	3.95 [2.30, 5.60]	0.427
	Akkermansia	3.28 [1.33, 5.24]	2.77 [0.79, 4.74]	0.370
	Roseburia	3.39 [2.39, 4.39]	1.76 [1.19, 2.33]	0.142
	Erysipelatoclostridium	0.28 [0.03, 0.54]	2.4 [1.05, 3.75]	**0.036**
	Odoribacterium	0.51 [0.35, 0.68]	0.31 [0.15, 0.47]	**0.036**
	Dialister	0.21 [0.11, 0.31]	0.15 [0.03, 0.26]	0.341
	Bifidobacterium	0.24 [0.08, 0.40]	0.10 [-0.01, 0.20]	0.252
Methionine	*Bacteroides*	31.54 [26.73, 36.36]	26.88 [22.51, 31.24]	0.432
	Faecalibacterium	8.01 [5.97, 10.05]	9.91 [7.12, 12.69]	0.699
	Escherichia/Shigella	5.61 [3.43, 7.79]	4.24 [1.89, 6.59]	0.432
	Akkermansia	3.92 [1.89, 5.96]	1.93 [0.14, 3.73]	0.699
	Roseburia	2.83 [1.93, 3.73]	2.25 [1.53, 2.97]	0.920
	Erysipelatoclostridium	1.32 [0.33, 2.31]	1.39 [0.32, 2.46]	0.962
	Odoribacterium	0.28 [0.19, 0.38]	0.56 [0.33, 0.78]	0.699
	Dialister	0.14 [0.06, 0.21]	0.23 [0.09, 0.37]	0.920
	Bifidobacterium	0.26 [0.09, 0.42]	0.06 [0.01, 0.11]	0.432

1. The median of the nutrient intake was used to dichotomize low versus high nutrient intake.
